# Urinary N-acetyltyramine-O,β-glucuronide in Persons with Onchocerciasis-Associated Epilepsy

**DOI:** 10.3390/pathogens9030191

**Published:** 2020-03-05

**Authors:** An Hotterbeekx, Alfred Dusabimana, Michel Mandro, Germain M Abhafule, Wonya’Rossy Deogratias, Joseph N. Siewe Fodjo, Steven Abrams, Robert Colebunders

**Affiliations:** 1 Global Health Institute, University of Antwerp, B-2610 Wilrijk, Belgium; alfred.dusabimana@uantwerpen.be (A.D.); JosephNelson.SieweFodjo@uantwerpen.be (J.N.S.F.); steven.abrams@uantwerpen.be (S.A.); robert.colebunders@uantwerpen.be (R.C.); 2 Provincial Health Division Ituri, Ministry of Health, 185 DRC 57 Ituri, Congo; Michel.MandroNdahura@student.uantwerpen.be (M.M.); abhafule@gmail.com (G.M.A.); deogratiasrossy@yahoo.fr (W.D.); 3 Interuniversity Institute for Biostatistics and statistical Bioinformatics, Data Science Institute, UHasselt, B-3590 Diepenbeek, Belgium

**Keywords:** *Onchocerca volvulus*, N-acetyltyramine-O,β-glucuronide (NATOG), epilepsy, Africa, biomarker, urine

## Abstract

We investigated urinary N-acetyltyramine-O,β-glucuronide (NATOG) levels as a biomarker for active *Onchocerca volvulus* infection in an onchocerciasis-endemic area in the Democratic Republic of Congo with a high epilepsy prevalence. Urinary NATOG was measured in non-epileptic men with and without *O. volvulus* infection, and in *O. volvulus-*infected persons with epilepsy (PWE). Urinary NATOG concentration was positively associated with microfilarial density (*p* < 0.001). The median urinary NATOG concentration was higher in PWE (3.67 µM) compared to men without epilepsy (1.74 µM), *p* = 0.017; and was higher in persons with severe (7.62 µM) compared to mild epilepsy (2.16 µM); *p* = 0.008. Non-epileptic participants with and without *O. volvulus* infection had similar NATOG levels (2.23 µM and 0.71 µM, *p* = 0.426). In a receiver operating characteristic curve analysis to investigate the diagnostic value of urinary NATOG, the area under the curve was 0.721 (95% CI: 0.633–0.797). Using the previously proposed cut-off value of 13 µM to distinguish between an active *O. volvulus* infection and an uninfected state, the sensitivity was 15.9% and the specificity 95.9%. In conclusion, an *O. volvulus* infection is associated with an increased urinary NATOG concentration, which correlates with the individual parasitic load. However, the NATOG concentration has a low discriminating power to differentiate between infected and uninfected individuals.

## 1. Introduction

The filarial nematode *Onchocerca volvulus* is transmitted by blackflies (*Simuliidae*) and causes skin disease (itching and rash), eye disease (progressive loss of vision), and epilepsy (onchocerciasis-associated epilepsy (OAE)) [1]. A recent study showed that children with a high microfilarial (mf, larval stage) density (>200 mf/skin snip) were 28 times more likely to develop epilepsy as compared to children without mf [2]. OAE is an important public health problem, since it is estimated that there are approximately 100,000–400,000 persons with OAE in Africa, which could have been prevented through improved onchocerciasis control [3].

Currently, elimination programs are ongoing in onchocerciasis-endemic regions through the mass distribution of ivermectin (community-directed treatment with ivermectin (CDTI)) [4,5]. Ivermectin kills the microfilariae and temporarily represses the production of new microfilariae by the female worms, albeit without killing the adult worms [6]. Onchocerciasis is diagnosed through the detection of microfilariae in skin snips. However, obtaining skin snips requires an invasive, slightly painful procedure and is therefore not accepted everywhere, especially in low-endemic regions. Alternatively, exposure to *O. volvulus* can be estimated by means of a rapid diagnostic test (RDT) detecting IgG4 antibodies against the OV16 antigen of *O. volvulus* [7]. The OV16 RDT was reported to have a specificity of 98–100% and a sensitivity of 76.5%, 81% or 90%, depending on the population [7,8]. A disadvantage of the aforementioned test is that it does not differentiate between active infection and past exposure and that it does not provide information about infection load. 

Previous research exploring the metabolomic profile of *O. volvulus-*infected individuals living in villages in Ghana and Cameroon, revealed that N-acetyltyramine-O,β-glucuronide (NATOG) was enriched in the urine of *O. volvulus-*infected individuals [9]. NATOG is the inactivated form of the *O. volvulus* neurotransmitter tyramine, which is excreted in the urine of infected individuals [9,10]. Since the discovery of NATOG, several studies have tried to determine a cut-off value for urinary NATOG concentration to diagnose active *O. volvulus* infection [9,10,11,12]. However, the results of these studies are conflicting. Therefore, the urinary NATOG threshold indicating an active *O. volvulus* infection remains unknown. Furthermore, the correlation between urinary NATOG concentration and infection load is unclear. 

In this study, we aimed to further explore the potential of urinary NATOG as a biomarker for *O. volvulus* infection in ivermectin naive individuals with and without epilepsy in an onchocerciasis-endemic area of the Democratic Republic of Congo (DRC), where an epilepsy prevalence of 4.6% was documented in 2016 [13]. In addition, we investigated the association between urinary NATOG concentration and skin mf density in persons with epilepsy. 

## 2. Materials and Methods

### 2.1. Study Population and Sample Collection

The study was performed in the Logo health zone, Ituri province, DRC, in onchocerciasis-endemic villages where ivermectin was never distributed. Ethical approval was obtained by the ethical committee of the university hospital of Antwerp (May 24, 2017, B300201733011) and the ethical committee of the university of Kinshasa (February 28, 2018, ESP/CE/013/2018). Men older than 20 years, having no epileptic seizures and living in these villages for more than 10 years, were asked to participate in a rapid epidemiological mapping of onchocerciasis (REMO) to determine the degree of onchocerciasis-endemicity in the area. They were examined for the presence and number of palpable nodules and tested for exposure to *O. volvulus* using the OV16 IgG4 rapid diagnostic test (OV16 RDT, SD Bioline Onchocerciasis IgG4 rapid test, Abbott Standard Diagnostics, Inc., Yongin, Republic of Korea). Twenty men with evidence of *O. volvulus* infection (OV16 RDT positive with the presence of at least one nodule) and 19 men without evidence of *O. volvulus* infection (OV16 RDT negative and no nodules) were asked to provide a urine sample. Skin snip testing was not performed on these subjects.

In addition, persons with epilepsy (PWE) and with parasitological and/or serological evidence of *O. volvulus* infection were recruited during a clinical trial investigating the effect of ivermectin on seizure frequency [14]. PWE were randomized in two groups according to a 1:1 ratio, with one group receiving phenobarbital and an ivermectin dose of 150 µg/kg and the other group receiving only phenobarbital [15]. After informed consent was obtained, participants were interviewed to document their medical history and a physical examination was performed, including a neurological assessment. Participants were examined for the presence of nodules, but a nodule count was not recorded. Skin snips were obtained from the left and right iliac crests to determine the mf density; the OV16 RDT was used to detect *O. volvulus* antibodies and participants provided a urine sample. PWE having two or more seizures per month were classified as having a severe form of epilepsy whereas those with less than two seizures per month were considered to have a mild form of epilepsy. The PWE had not received antiepileptic drug treatment within the last two weeks before enrolment in this study. Four months after enrolment, a follow-up clinical examination was performed, skin snip testing was repeated, and a second urine sample was obtained.

Persons without epilepsy were considered to have an active *O. volvulus* infection if they were OV16 RDT positive, had never received ivermectin and had palpable nodules, in the absence of skin snip results. Ivermectin-naive PWE were considered to have active infection when mf was present in skin snips, or if they had a positive OV16 RDT, with no mf but with nodules. Ivermectin-naive PWE with a positive OV16 test with no mf in their skin snips and no nodules were not considered to have an active infection, but a pre-patent infection (whereby adult females are not yet producing mf). 

### 2.2. Mass Spectrometry Analysis

NATOG concentrations were determined in urine samples by liquid chromatography tandem mass spectrometry (LC-MS/MS) at Janssen Global Public Health (Beerse, Belgium), as described earlier [12]. Quality control and calibration samples were prepared as described before [12] and a standard curve (1–100 µM) was constructed by spiking stock solutions of NATOG to 25-fold diluted urine. The LOQ was defined at 0.7 µM. Urine samples were diluted 25-fold in MilliQ-water and were first separated using a UPLC (Acquity UPLC; HSS T3 column; 1.8 µm, 2.1 × 50 mm, Waters Corporation) before analysis by electrospray ionisation (ESI) with triple quadrupole MS/MS (API 4000, AB Sciex). Data were collected using SRM in positive ESI mode with Q1 Mass 356.1 Da and Q3 Mass 180.1 Da. The calibration data were used to estimate a linear regression curve, that was subsequently considered to determine the NATOG concentrations of the collected urine samples. 

### 2.3. Statistical Analysis

Medians and interquartile ranges (IQRs) were used to describe continuous variables, whereas categorical variables were characterized using absolute and relative frequencies. The correlation between urinary NATOG concentration and the number of nodules was examined using a Kendall’s tau-b rank correlation coefficient. The association between mf density and urinary NATOG concentration was assessed using a Quasi-Poisson regression model while accounting for the dependence between pre- and post-treatment measurements on the same subjects using a generalized estimating equations (GEE) approach [16]. A logistic regression model with parameter estimation using a GEE approach was considered to assess the relationship between urinary NATOG concentration and active *O. volvulus* infection in ivermectin-naive PWE. Receiver operating characteristic (ROC) curves were constructed to illustrate the diagnostic ability of classifying persons with and without *O. volvulus* infection in two groups based on their observed urinary NATOG concentrations when the discrimination threshold is varied. Two-sided *p*-values <0.05 were considered statistically significant. Analyses were performed using SAS 9.4, SAS Institute Inc. and R, version 3.6.1. 

### 2.4. Ethical Considerations and Informed Consent

The study was approved by the Ethics Committee of the School of Health of the University of Kinshasa and the University of Antwerp, Antwerp, Belgium. All eligible candidates provided written informed consent before enrolment into the study.

## 3. Results 

### 3.1. Urinary NATOG Concentration in Ivermectin-Naive men without Epilepsy

**Figure 1:**
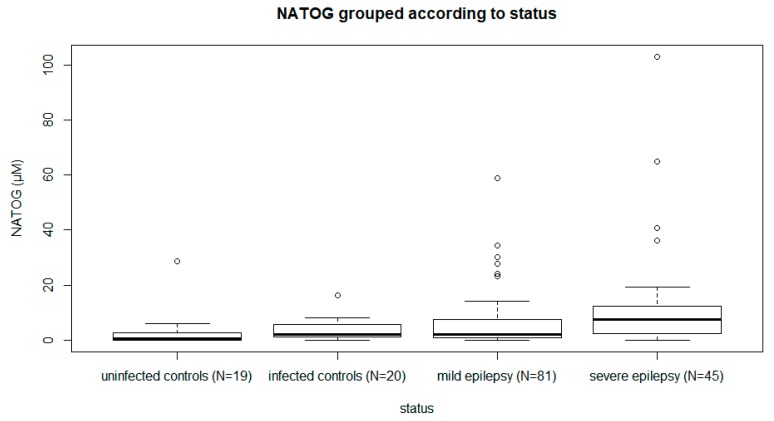
Urinary N-acetyltyramine-O,β-glucuronide (NATOG) concentrations in persons with and without epilepsy living in onchocerciasis endemic villages in the Logo health zone, Ituri province, Democratic Republic of Congo (DRC).

Twenty *O. volvulus-*infected and 19 uninfected ivermectin-naive men without epilepsy participated in the study. Infected men were older than uninfected men (median age 46 (IQR: 35–59) and 33 (IQR: 23–46), respectively). The median urinary NATOG concentration was not different in *O. volvulus*-infected (2.23 µM (IQR: 1.31–5.58)) compared to non-infected men (0.71 µM (IQR: 0.00–2.78), *p* = 0.426, Figure 1). Furthermore, the NATOG concentration was positively correlated with the number of nodules (Kendall’s Tau-b coefficient **=** 0.321, *p* = 0.014).

### 3.2. Urinary NATOG Concentration in Ivermectin-Naive Persons with Epilepsy

A total of 134 PWE with a positive skin snip and/or a positive OV16 RDT participated in the study. Of those, 117 (87%) had active *O. volvulus* infection. Their median age was 24 years, and 68 (53.5%) of them were male. The median mf density of the PWE was 10 mf/skin snip (IQR: 0–59), and the median urinary NATOG concentration was 3.67 µM (IQR: 1.24–8.55). The median seizure frequency was two seizures per month (IQR: 0–4). The median urinary NATOG concentration was higher in PWE (3.67 µM (IQR: 1.24–8.55)) compared to men without epilepsy (1.74 µM (IQR: 0.00–3.14), median test *p* = 0.017) and higher in persons with severe epilepsy (7.62 µM (IQR: 2.52–15.5)) compared to those with mild epilepsy (2.17 µM (IQR: 1.00–7.59), median test *p* = 0.008) (Table 1, Figure 1). Similarly, the median mf density was higher (19.5 mf/skin snip (IQR: 2–84)) in persons with severe epilepsy compared to persons with mild epilepsy (5 mf/skin snip (IQR: 0–40), median test *p* = 0.041) and a higher proportion of persons with severe epilepsy (82%) had active *O. volvulus* infection compared to persons with mild epilepsy (67%, chi-square *p* = 0.029).

Furthermore, there was an increasing trend in median NATOG concentration from uninfected to infected men without epilepsy (*p* = 0.028), and from persons with mild to severe epilepsy (*p* = 0.004) (Figure 1). A logistic regression model showed a significant association between urinary NATOG concentration and active *O. volvulus* infection before (odds ratio (OR): 3.116, 95% CI: 1.970–4.930; *p* < 0.001) and after (OR: 1.343, 95% CI: 1.023–1.764, *p* = 0.034) ivermectin treatment (Table 2). Moreover, mf density was significantly associated with urinary NATOG concentration before and after ivermectin administration (Table 3).

### 3.3. Urinary NATOG Concentration of PWE, Four Months after Ivermectin or without Ivermectin Treatment 

Ninety-two (68%) of the 134 PWE received ivermectin. The median mf density for the group of PWE who received ivermectin was 8.75 mf/skin snip (IQR: 0.00–77.50) before and 0 mf/skin snip (IQR: 0.00–1.50) four months after treatment, a median reduction of 100%. The median of urinary NATOG concentration reduced from 3.67 (IQR: 1.21–8.49) to 1.55 µM (IQR: 0.00–3.22), a median reduction of 75.1% (Table 4).

Forty-two (32%) individuals did not receive ivermectin. The median mf density reduced from 12.5 mf/skin snip (IQR: 0.5–55.5) at enrollment to 0.5 mf/skin snip (IQR: 0–20) in the follow-up sample four months later, a median reduction of 83%, while the median of urinary NATOG concentration reduced from 3.58 (IQR: 1.42–9.01) to 1.65 µM (IQR: 0–5.19), a median reduction of 64.9% (Table 4). 

### 3.4. Urinary NATOG as a Biomarker for Active O. volvulus Infection

**Figure 2:**
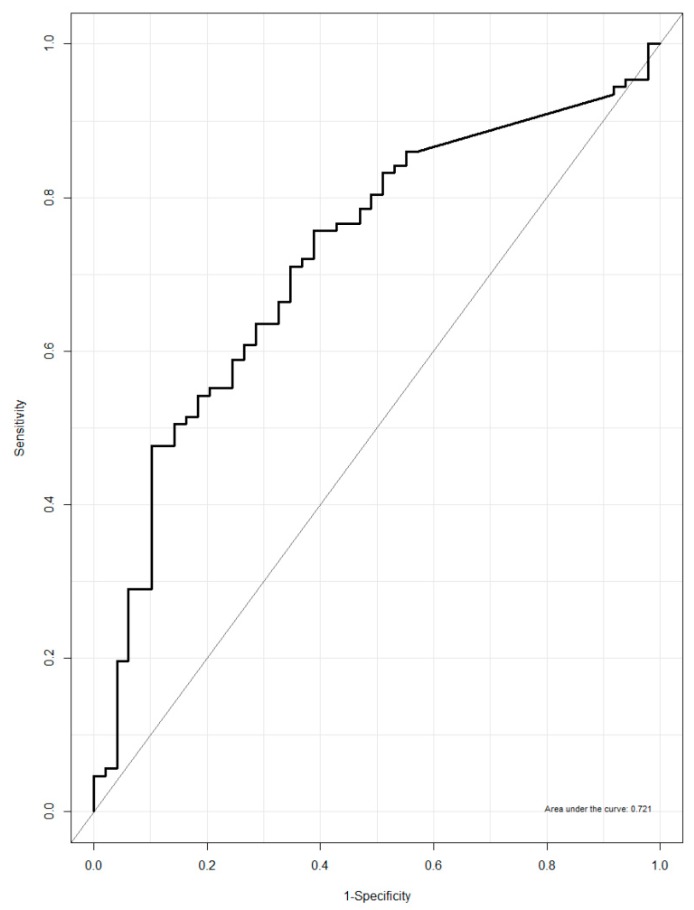
Receiver operating characteristic (ROC) curve urinary NATOG concentration in ivermectin-naive persons with and without epilepsy.

The discriminative power of urinary NATOG concentration to discriminate between active *O. volvulus* infected and non-infected individuals was found to be low, with an area under the curve (AUC) of 0.721 (95% CI: 0.633–0.797, Figure 2). Furthermore, using the previously proposed cut-off value of 13 µM, 17 individuals were correctly classified as having active infection and 47 individuals were correctly classified as not having active infection, leading to a sensitivity of 15.9% and a specificity of 95.9% (Table 5). 

Using the previously proposed NATOG cut-off value of 13 µM for the diagnosis of active *O. volvulus* infection and an uninfected state [10], the sensitivity of urinary NATOG was 15.9% and specificity was 95.9% (Table 5).

## 4. Discussion

This is the first study investigating the association between urinary NATOG concentration and *O. volvulus* mf density and the effect of ivermectin treatment on urinary NATOG concentrations in individuals with OAE. The urinary NATOG concentration decreased after ivermectin treatment, with a corresponding drop in mf density. Furthermore, we observed higher mf densities and urinary NATOG concentrations in PWE compared to persons without epilepsy, and an increasing trend in NATOG concentration from uninfected individuals (lowest concentration) to infected individuals, persons with mild epilepsy, and persons with severe epilepsy (highest concentration). However, there was no difference in urinary NATOG between infected and uninfected people without epilepsy. 

Both the urinary NATOG concentration and skin mf density decreased in the ivermectin-treated and untreated PWE. However, the decrease in mf density (100%) in the treated group compared to the untreated group (83%) was significant, while the decrease in urinary NATOG concentration between both groups was not significant. The explanation for the decrease in mf density and urinary NATOG concentration in PWE that were not treated with ivermectin is unclear. After performing skin snips, all PWE were started on phenobarbital. Currently, the effect of phenobarbital on *O. volvulus* is unknown, but this anti-epileptic drug acts as a gamma-aminobutyric acid (GABA) A receptor subunit agonist [17]. GABA-gated chloride channels are required for *O. volvulus* locomotion and are a drug target for ivermectin [18]. Therefore, one explanation could be that phenobarbital may have reduced mf densities or motility, leading to reduced emergence from the skin snip before counting, potentially by acting on the parasite GABA-receptor subunits. However, it cannot be excluded that pre-analytic differences in urine collection or differences in skin snip collection influenced the results.

The average urinary NATOG concentration in individuals with active infection in our study was 8.9 µM, which is close to the 8.4 µM observed in Guatemalan samples in a study by Globisch et al. [9] (Table 6). This concentration is below the 13 µM threshold required to identify active *O. volvulus* infection in Africa, proposed by Globisch et al. This high threshold was proposed by Globisch et al. because, in a study in Ghana, high urinary NATOG concentrations were observed in *O. volvulus* uninfected endemic African controls (Table 6) [9,10]. However, the diagnosis of uninfected controls was mainly based on the absence of nodules. In only a limited number of participants were skin snips obtained, and mf densities were not taken into account. Another explanation for the elevated urinary NATOG concentrations in the participants from Ghana might be co-infections with other nematodes, such as *Mansonella perstans* and/or *Loa Loa*. Indeed, urinary NATOG concentrations in *O. volvulus* non-infected individuals with other nematodes was, on average, 9.29 µM [10]. Eight individuals with an *O. volvulus*, *Loa. loa* and *M. perstans* co-infection had very high NATOG concentrations 100.5 ± 33.5 µM [10]. None of the PWE in Ituri were *Loa loa-*infected, but co-infection with *M. perstans* was not assessed. Globisch et al suggested that the lower urinary NATOG in the Guatemala samples compared to their samples obtained in Ghana and Cameroon could be explained by the genetic diversity of *O. volvulus*. A more likely explanation is that the mf densities in Guatemala were lower than those in Ghana and Cameroon because of previous ivermectin exposure. In fact, very low urinary NATOG concentrations (1.06 µM) were also detected in another population in Ghana, investigated by Lagatie et al. In the latter study, low mf densities (0–10 mf/mg skin) most likely resulting from previous ivermectin use, may explain the low NATOG concentrations observed [12]. Indeed, the urinary NATOG concentrations and skin mf densities in the study by Lagatie et al. were similar to those observed in our study participants after ivermectin treatment (Table 6). Recently, a lateral flow immunoassay (LFIA) test was developed to detect urinary NATOG [19]. In a small study, this test accurately diagnosed 23 (85%) of the 27 African samples tested, with a cut-off concentration set at 25 µM [19]. However, the authors of the above-mentioned study did not provide detailed information about mf densities. 

Our study has several limitations. First, only a limited number of persons without epilepsy were recruited and their mf densities were not determined. It is possible that the low urinary NATOG concentrations in persons without epilepsy was due to a low degree of infection, and may not necessarily reflect background NATOG levels in uninfected individuals. The number of nodules were not counted in PWE and only two skin snips were performed per person. Therefore, an active infection with a low mf density may have been missed in certain individuals.

In conclusion, our study confirms that *O. volvulus* infection is associated with an increased urinary NATOG concentration and that this concentration depends on the individual mf density. However, due to a large inter- individual and inter-regional variability in urinary NATOG concentrations, and an overlap between infected and uninfected individuals, there is a low discriminative power for urinary NATOG concentration to differentiate between infected and uninfected individuals.

## Figures and Tables

**Table 1:** Urinary NATOG levels and characteristics of ivermectin-naïve and *O. volvulus-*infected individuals with mild and severe epilepsy.

	*O. volvulus*-infected ^a^ with Mild Epilepsy (n = 81)	*O. volvulus*-infected ^a^ with Severe Epilepsy (n = 45)	*P*-Value ^b^
Male: n (%)	40 (49%)	27 (60%)	0.269
Age in years: median (IQR)	24 (18–32)	22 (16–28)	0.578
Seizures per month: median (IQR)	1 (0–2)	4 (4–10)	<0.001
Skin mf density: median (IQR)	5 (0–40)	19.5 (2–84)	0.041
Positive skin snip: n (%)	54 (67%)	37 (82%)	0.029
NATOG (µM): median (IQR)	2.17 (1–7.59)	7.62 (2.52–15.5)	0.008

^a^ Skin-snip-positive and/or OV16 rapid diagnostic test (RDT)-positive in ivermectin naive individuals; ^b^ Median test for continuous variables and Fisher’s exact test for categorical variables; IQR: inter-quartile range; mf: microfilarial.

**Table 2:** Odds ratios and 95% confidence intervals for the effect of different covariates on the probability of active *O. volvulus* infection in ivermectin-naive persons with epilepsy based on a logistic regression model with generalized estimating equations (GEE) approach.

Effect	OR	95% CI		*P*-Value
Log-transformed urinary NATOG before ivermectin treatment	3.116	1.970	4.930	<0.001
Log-transformed urinary NATOG after ivermectin treatment	1.343	1.023	1.764	0.034
Age	1.003	0.980	1.026	0.823
Female vs. male	1.124	0.609	2.074	0.708
Number of seizures in last 2 months prior to ivermectin treatment	1.036	0.989	1.085	0.132

OR: odds ratio, CI: confidence interval.

**Table 3:** Multiplicative effects (estimate) of covariates on the microfilarial density with corresponding 95% confidence intervals and p-values based on a Quasi-Poisson regression model with GEE.

Effect	Estimate	95% CI		*P*-Value
Log-transformed urinary NATOG before ivermectin treatment	2.344	1.895	2.900	<0.0001
Log-transformed urinary NATOG after ivermectin treatment	1.560	1.169	2.081	0.003
Age	1.003	0.984	1.023	0.726
Female vs. male	1.185	1.066	1.318	0.521
Number of seizures last 2 months before ivermectin	1.025	0.974	1.079	0.344

OR: odds ratio, CI: confidence interval.

**Table 4:** Urinary NATOG concentration and characteristics of persons with epilepsy who received and did not receive ivermectin.

	*O. volvulus*-Infected PWE ^a^ (n = 134)		
	Received Ivermectin (n = 92)	Did Not Receive Ivermectin (n = 42)	*P*-Value ^b^
Male: n (%)	45 (49%)	23 (55%)	0.374
Age in years: median (IQR)	24 (18–32)	22 (17–29)	0.272
OV16 RDT-positive: n (%)	58 (63%)	27 (64%)	0.825
Skin mf density at baseline: median (IQR)	8.75 (0–77.5)	12.5 (0.5–55.5)	0.536
Positive skin snip at baseline*: n (%)	63 (70.8%)	39 (76.3%)	0.665
Skin mf density at follow-up: median (IQR)	0 (0–1.5)	0 (0–20)	0.081
Positive skin snip at follow-up: n (%)	28 (31.5%)	19 (47.5%)	0.113
Mf % reduction: median (IQR)	100% (92.3–100.0)	83.1% (66.7–100.00)	0.014
NATOG (µM) at baseline: median (IQR)	3.7 (1.2–8.5)	3.6 (1.4–9.0)	0.983
NATOG (µM) at follow-up: median (IQR)	1.6 (0–3.22)	1.7 (0–5.19)	0.654
NATOG (µM) % reduction: median (IQR)	75.1% (18.6–100.0)	64.9% (8.0–83.1)	0.101
Seizures per month at baseline: median (IQR)	2.0 (0.0–2.5)	4.0 (2.0–4.0)	<0.001

^a^ Skin-snip-positive and/or OV16-positive; ^b^ Median test for continuous variables and Fisher’s exact test for categorical variables; * seven missing values; IQR: Inter quartile range; mf: microfilariae; Baseline: initial screening; Follow-up: 4 months after initial screening.

**Table 5:** Sensitivity and specificity of urinary NATOG in diagnosing active onchocerciasis (Cut-off: 13 µM).

Classified by NATOG as:	*O. volvulus* Infection		Total
	No active Infection	Active Infection	
Negative (n)	47	90	**137**
Positive (n)	2	17	**19**
**Total**	**49**	**107**	**156**

**Table 6:** Urinary NATOG concentrations in populations from different countries across different studies.

Study	Country	Population (n)	NATOG concentration (µM)				Remarks
			Average ± SEM	Median	Min	Max	
Globisch et al. (2013)	Ghana + Cameroon	*O. volvulus* positive (81)	36.9 ± 4	na	na	na	*O. volvulus* infection diagnosed by nodule palpation or skin snips, but mf densities not reported
	Ghana + Cameroon	Uninfected control (16)	7.0 ± 2.7	na	na	na	
	North America	Non-endemic control (17)	1.1 ± 0.2	na	na	na	
	Guatemala	*O. volvulus* positive (20)	8.4 ± 1.6	na	na	na	
	Ghana + Cameroon	Lymphatic filariasis (23)	4.2 ± 0.7	na	na	na	
	Ghana	*O. volvulus* positive, 20 months after doxycyclin (24)	9.5 ± 1.7	na	na	na	
	Ghana	*O. volvulus* positive, 20 months after placebo (14)	33.5 ± 10.7	na	na	na	
Globisch et al. (2017)	Ghana + Cameroon	*O. volvulus* positive (145)	42.8 ± 3.7	29.3	0.9	276	*O. volvulus* infection diagnosed by nodule palpation or skin snips, but mf densities not reported
	Ghana + Cameroon	Uninfected control (118)	6.4 ± 0.7	3.6	0.2	39.6	
	Ghana + Cameroon	*L. loa* infection (100)	14.7 ± 2.5	6.8	0.4	175.6	
	Ghana + Cameroon	*M. perstans* infection (25)	13.6 ± 2.5	11.4	0.3	46.4	
	Ghana + Cameroon	*L. loa* + *M. perstans* infection (3)	6.0 ± 2.7	6.5	1	10.4	
	Ghana + Cameroon	*O. volvulus* + *L. loa* infection (21)	16.6 ± 2.8	13.8	0.8	41.1	
	Ghana + Cameroon	*O. volvulus* + *M. perstans* infection (29)	29.2 ± 4.8	17.1	2.2	92.8	
	Ghana + Cameroon	*O. volvulus + L. loa + M. perstans* infection (8)	100.5 ± 33.5	66.4	4.7	246.5	
Lagatie et al. (2016)	Ghana	*O. volvulus* positive (98)	1.06 ± 0.16	na	na	na	*O. volvulus* infection diagnosed by nodule palpation, skin snips and OV16 RDT; 82% previously received ivermectin; in 89% no mf in skin snips
	Ghana	Endemic control (50)	0.95 ± 0.8	na	na	na	
	Ghana	Lympathic filariasis (51)	0.99 ± 0.17	na	na	na	
	Europe	Non-endemic control (18)	0.66 ± 0.18	na	na	na	
Current study	DRC	All active *O. volvulus* infected (117)	9.7 ± 1.4	5.3	0	103	*O. volvulus* infection diagnosed by nodule palpation, skin snips and OV16 RDT
	DRC	*O. volvulus* uninfected (55)	3.2 ± 0.9	1.3	0	34.5	
	DRC	*O. volvulus* negative no epilepsy (19)	3 ± 1.5	0.71	0	28.6	
	DRC	*O. volvulus* infected no epilepsy (20)	3.7 ± 0.9	2.23	0	116.3	
	DRC	*O. volvulus* infected with mild epilepsy (81)	6.1 ± 1.2	2.17	0	58.9	
	DRC	*O. volvulus* infected with severe epilepsy (45)	12 ± 3	7.62	0	103	
	DRC	*O. volvulus* infected with epilepsy, before ivermectin (134)	8.2 ± 1.3	3.67	0	103	
	DRC	*O. volvulus* infected with epilepsy, after ivermectin (92)	3 ± 0.6	1.55	0	33.8	

na: not available; SEM: standard error of the mean; mf: microfilarial.

## References

[1] Colebunders R., Nelson Siewe F.J., Hotterbeekx A. (2018). Onchocerciasis-Associated Epilepsy, an Additional Reason for Strengthening Onchocerciasis Elimination Programs. Trends Parasitol..

[2] Chesnais C.B., Nana-Djeunga H.C., Njamnshi A.K., Lenou-Nanga C.G., Boulle C., Bissek A.Z., Kamgno J., Colebunders R., Boussinesq M. (2018). The temporal relationship between onchocerciasis and epilepsy: A population-based cohort study. Lancet Infect. Dis..

[3] Colebunders R., Irani J., Post R. (2016). Nodding syndrome—We can now prevent it. Int. J. Infect. Dis..

[4] Tekle A.H., Zoure H.G., Noma M., Boussinesq M., Coffeng L.E., Stolk W.A., Remme J.H. (2016). Progress towards onchocerciasis elimination in the participating countries of the African Programme for Onchocerciasis Control: Epidemiological evaluation results. Infect. Dis. Poverty.

[5] WHO (1995). Onchocerciasis and Its Control.

[6] Remme J.H. (2004). Research for control: The onchocerciasis experience. Trop. Med. Int. Health.

[7] Weil G.J., Steel C., Liftis F., Li B.W., Mearns G., Lobos E., Nutman T.B. (2000). A rapid-format antibody card test for diagnosis of onchocerciasis. J. Infect. Dis..

[8] Lipner E.M., Dembele N., Souleymane S., Alley W.S., Prevots D.R., Toe L., Boatin B., Weil G.J., Nutman T.B. (2006). Field applicability of a rapid-format anti-Ov-16 antibody test for the assessment of onchocerciasis control measures in regions of endemicity. J. Infect. Dis..

[9] Globisch D., Moreno A.Y., Hixon M.S., Nunes A.A., Denery J.R., Specht S., Hoerauf A., Janda K.D. (2013). Onchocerca volvulus-neurotransmitter tyramine is a biomarker for river blindness. Proc. Natl. Acad. Sci. USA.

[10] Globisch D., Eubanks L.M., Shirey R.J., Pfarr K.M., Wanji S., Debrah A.Y., Hoerauf A., Janda K.D. (2017). Validation of onchocerciasis biomarker N-acetyltyramine-O-glucuronide (NATOG). Bioorg. Med. Chem. Lett..

[11] Bennuru S., Cotton J.A., Ribeiro J.M., Grote A., Harsha B., Holroyd N., Mhashilkar A., Molina D.M., Randall A.Z., Shandling A.D. (2016). Stage-Specific Transcriptome and Proteome Analyses of the Filarial Parasite Onchocerca volvulus and Its Wolbachia Endosymbiont. mBio.

[12] Lagatie O., Njumbe Ediage E., Batsa Debrah L., Diels L., Nolten C., Vinken P., Debrah A., Dillen L., Silber S., Stuyver L.J. (2016). Evaluation of the diagnostic potential of urinary N-Acetyltyramine-O,beta-glucuronide (NATOG) as diagnostic biomarker for Onchocerca volvulus infection. Parasit. Vectors.

[13] Lenaerts E., Mandro M., Mukendi D., Suykerbuyk P., Dolo H., Wonya'Rossi D., Ngave F., Ensoy-Musoro C., Laudisoit A., Hotterbeekx A. (2018). High prevalence of epilepsy in onchocerciasis endemic health areas in Democratic Republic of the Congo. Infect. Dis. Poverty.

[14] Mandro M., Siewe Fodjo J.N., Mukendi D., Dusabimana A., Menon S., Haesendonckx S., Lokonda R., Nakato S., Nyisi F., Abhafule G. (2020). Ivermectin as an adjuvant to anti-epileptic treatment in persons with onchocerciasis-associated epilepsy: A randomized proof-of-concept clinical trial. PLoS Negl. Trop. Dis..

[15] Colebunders R., Mandro M., Mukendi D., Dolo H., Suykerbuyk P., Van Oijen M. (2017). Ivermectin Treatment in Patients With Onchocerciasis-Associated Epilepsy: Protocol of a Randomized Clinical Trial. JMIR Res. Protoc..

[16] Sen P.K. (1968). Estimates of the Regression Coefficient Based on Kendall's Tau. J. Am. Stat. Assoc..

[17] Lewis C.B., Adams N. (2019). Phenobarbital. StatPearls.

[18] Wolstenholme A.J., Rogers A.T. (2005). Glutamate-gated chloride channels and the mode of action of the avermectin/milbemycin anthelmintics. Parasitology.

[19] Shirey R.J., Globisch D., Eubanks L.M., Hixon M.S., Janda K.D. (2018). Noninvasive Urine Biomarker Lateral Flow Immunoassay for Monitoring Active Onchocerciasis. ACS Infect. Dis..

